# Sex-dependent association analysis between serum uric acid and spontaneous hemorrhagic transformation in patients with ischemic stroke

**DOI:** 10.3389/fneur.2023.1103270

**Published:** 2023-03-03

**Authors:** Ye Tang, Ming-Su Liu, Chong Fu, Guang-Qin Li

**Affiliations:** Department of Neurology, The First Affiliated Hospital of Chongqing Medical University, Chongqing, China

**Keywords:** uric acid, hemorrhagic transformation, reperfusion therapy, admission time, male

## Abstract

**Objective:**

The association between serum uric acid (UA) and spontaneous hemorrhagic transformation (HT) has been seldom studied, and the role of UA in spontaneous HT remains unclear. This study aims to investigate the sex-dependent association between UA and spontaneous HT in patients with ischemic stroke.

**Method:**

We retrospectively included patients with ischemic stroke in a tertiary academic hospital between December 2016 and May 2020. Patients were included if they presented within 24 h after the onset of symptoms and did not receive reperfusion therapy. Spontaneous HT was determined by an independent evaluation of neuroimaging by three trained neurologists who were blinded to clinical data. A univariate analysis was performed to identify factors related to spontaneous HT. Four logistic regression models were established to adjust each factor and assess the association between UA and spontaneous HT.

**Results:**

A total of 769 patients were enrolled (64.6% were male patients and 3.9% had HT). After adjusting the confounders with a *P* < 0.05 (model A) in the univariate analysis, the ratio of UA and its interquartile range (RUI) was independently associated with spontaneous HT in male patients (OR: 1.85; 95% CI: 1.07–3.19; *P* = 0.028), but not in female patients (OR: 1.39; 95% CI: 0.28–6.82; *P* = 0.685). In models B–D, the results remain consistent with model A after the adjustment for other potential confounders.

**Conclusions:**

Higher serum UA was independently associated with a higher occurrence of spontaneous HT in male patients who were admitted within 24 h after the stroke onset without receiving reperfusion therapy.

## Introduction

Spontaneous hemorrhagic transformation (HT) is defined as the blood stain of an infarcted cerebral area formed by the overflow of red blood cells and other blood components from blood vessels to the infarcted brain tissue, which is a part of the natural course of ischemic stroke and a crucial complication of treatment (1). Spontaneous HT occurs in ~13–43% of patients with ischemic stroke, and parenchymal hematoma is a critical factor in poor outcomes (2). Thus, it is important to identify the factors that determine the occurrence of HT. However, the pathophysiological mechanism of spontaneous HT remains uncertain.

Uric acid (UA) is an endogenous antioxidant produced by purine metabolism (3, 4). If the antioxidant substances are abundant, UA will show antioxidant properties. If there are more pro-oxidant substances, it will show pro-oxidant properties (5). In patients with acute ischemic stroke, oxygen free radicals will be produced after tissue ischemia–reperfusion, and UA presents antioxidant or pro-oxidant properties depending on the surrounding substances. It has been demonstrated that the dose–response relationship between UA and HT and higher UA was independently associated with a lower incidence of HT. On the contrary, higher UA levels are reported to be associated with a lower incidence of HT in different settings (6). An examination of UA is widely available in almost all clinical settings. For these reasons, UA may be a protective factor for spontaneous HT. Therefore, the critical clinical significance of the relationship between UA and spontaneous HT is a topic of research interest.

Nevertheless, reperfusion injury and blood–brain barrier damage after infarction are considered the two major causes of spontaneous HT, and the reactive oxygen species (ROS)-mediated oxidative stress response has an important role in these two mechanisms (7). Many studies have explored the role of thrombolysis in HT occurrence (1, 7, 8). Although thrombectomy is not independently associated with spontaneous HT (9), given its mechanism of reperfusion therapy (e.g., thrombolysis or thrombectomy), the restoration of blood flow to the salvageable ischemic brain tissue is consistent with the aforementioned mechanism of spontaneous HT and the high incidence of spontaneous HT found by previous studies (10–12). None of these prior studies assessed spontaneous HT with respect to non-reperfusion strategies.

There is no consensus on the association between UA and spontaneous HT in patients with acute ischemic stroke. Studies of the relationship between UA levels and spontaneous HT are contradictory. Positive and negative in the male population or both positive in men and women have been described (8, 10, 13, 14). Furthermore, Brouns et al. found that UA changed with time in patients with stroke and exhibited a U-shaped curve in general, which decreased within 7 days after the stroke onset and then gradually increased to the baseline value (15). Few studies have explored the impact of UA levels in specific stroke subtypes and treatment strategies in the acute stage. UA levels are sex-dependent and are higher in males. Therefore, a sex-dependent explorative analysis was made using patients with acute ischemic stroke within 24 h after the stroke onset and who did not receive reperfusion therapy (thrombolysis or thrombectomy) after the onset to investigate whether UA was associated with spontaneous HT.

## Methods

### Population

We retrospectively reviewed the medical records of patients with ischemic stroke admitted to the Department of Neurology, the First Affiliated Hospital of Chongqing Medical University, from December 2016 to May 2020. For this analysis, the patients were included if they: (1) met the diagnostic criteria of acute ischemic stroke (AIS) in the Guidelines for Early Management of Patients With Acute Ischemic Stroke (2019) of the American Heart Association (AHA) (14), (2) were admitted within 24 h from the onset, (3) completed a serum UA test within 24 h after admission, and (4) had an initial neuroimaging scan [computed tomography (CT) scanning or magnetic resonance imaging (MRI)] within 24 h after admission and at least one follow-up neuroimaging scan within 7 days after admission. The exclusion criteria were as follows: (1) patients who received reperfusion therapy (thrombolysis or thrombectomy) after the onset, (2) patients with platelet abnormalities or coagulation dysfunction, (3) patients who received UA-lowering treatment within 1 month before admission, and (4) patients with intracranial arteriovenous malformation or tumor or head trauma. The First Affiliated Hospital of Chongqing Medical University Institutional Review Board approved this study. Written informed consent was obtained from participants or their legal representatives.

### Data collection

The clinical data were collected from each patient by two researchers: (1) demographic characteristics, such as age and sex; (2) medical histories, such as the history of smoking, alcohol consumption, hypertension, diabetes, dyslipidemia, and atrial fibrillation (AF); (3) clinical variables, such as National Institute of Health Stroke Scale (NIHSS), the Trial of ORG 10172 in Acute Stroke classification (TOAST), systolic blood pressure (SBP), diastolic blood pressure (DBP), and time from the stroke onset to admission; (4) laboratory tests, such as platelet count, activated partial thromboplastin time, serum UA, estimated glomerular filtration rate (eGFR), serum creatinine, low-density lipoprotein cholesterol (LDL-C), and hemoglobin A1c (HbA1c); (5) radiological characteristics, such as large hemispheric infarction (LHI) and spontaneous HT; and (6) treatment, such as anticoagulants, antiplatelet drugs, antihypertensive drugs, and antidiabetic drugs. Among them, eGFR was calculated by the serum creatinine level according to the formula of the Chronic Kidney Disease Epidemiology Collaboration (16). The cerebral infarct, of size >2/3 of MCA territory, was defined as LHI (17).

Serum UA concentration was tested by the enzymatic method (Roche Cobas C701) or the dry chemistry method (Ortho-Clinical Diagnostics). The diagnosis of spontaneous HT is based on the following criteria: abnormal hyperdensity within the area of low attenuation (CT) or abnormal hypointensity within the identified ischemic area (MRI) (13). The images were evaluated by two neurologists who were blinded to the patient's information. For inconsistent interpretations, the imaging was independently assessed by another senior neurologist, and the final diagnosis was determined on the principle of subordination of the minority to the majority. Furthermore, we classified spontaneous HT into four subtypes [type 1 and 2 hemorrhagic infarction (HI1 and 2) and type 1 and 2 parenchymal hemorrhage (PH1 and 2)] according to the European Cooperative Acute Stroke Study III (ECASS III) (18).

### Statistical analysis

Since there is no clinical significance of a 1-unit (1 μmol/L) change in UA in clinical practice, in this study, the ratio of UA and its interquartile range [RUI, male: RUI = (UA of individual male patient)/(IQR of UA in the male group), female: RUI = (UA of individual female patient)/(IQR of UA in the female group)] was used to replace UA in the statistical analysis, for increasing the practicability of the conclusions in clinical diagnosis and treatment. Continuous variables were expressed as the mean and standard deviation, and categorical variables were expressed as frequency and percentage. The comparison of continuous variables between groups was made by performing the *t*-test or Mann–Whitney U-test, whereas the comparison of categorical variables was made by performing the chi-square test or Fisher's exact test. In addition, the factors with a *P* < 0.05 in the univariate analysis and other factors that potentially could affect the study results were included in the subsequent logistic regression analysis. UA levels are lower in female patients, and a sex-dependent association between UA and cardiovascular disease was reported. Therefore, sex-dependent analysis was performed to investigate the impact of UA levels on HT occurrence. In total, four logistic regression models were built, and the association between UA and spontaneous HT was determined by dividing patients into two subgroups of male and female. These variables were chosen based on their known associations with the occurrence of HT, and their demonstrated link to HT in the logistical regression: Model A is adjusted for variables with a *P* < 0.05 in male (or female) patient subgroup univariate analysis; model B is adjusted for variables with a *P* < 0.05 in both subgroup univariate analysis; model C is adjusted for variables in model B, antiplatelet treatment, and anticoagulant treatment; model D is adjusted for variables in model C, smoking, alcohol consumption, systolic blood pressure, and eGFR. A *P* < 0.05 was considered statistically significant. Data analysis of the present study was performed by using SPSS Statistics Software (version 26.0; IBM Corporation) and GraphPad Prism (version 7.0; GraphPad Software Corporation).

## Results

**Figure 1:**
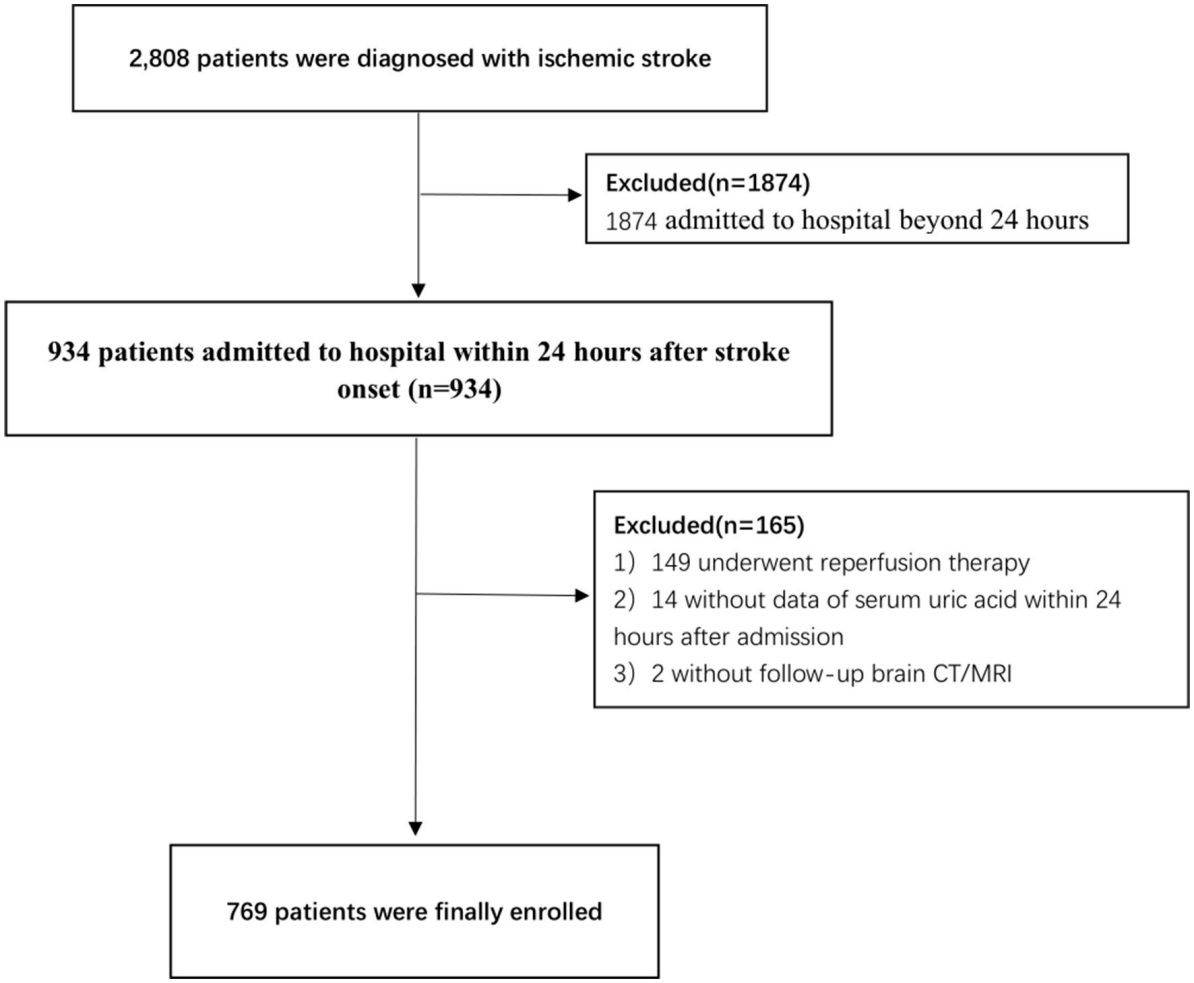
Flowchart of patients' selection.

A total of 769 patients were finally included in this study (Figure 1), of whom 64.6% were male patients, with a mean age of (66.9 ± 12.5) years and 30 (3.9%) had spontaneous HT. In this study, 70% of spontaneous HT was diagnosed by CT, 30% of spontaneous HT was diagnosed by MRI (T2WI and T1WI), 13.3% of patients with spontaneous HT performed SWI, and the result of SWI supports the diagnosis of CT/MRI. No patient with spontaneous HT was diagnosed by SWI alone. Among patients with spontaneous HT, one (3.3%) patient with PH2, seven (23.3%) patients with PH1, 13 (43.3%) patients with HI2, and nine (30.0%) patients with HI1 were identified. Compared with female patients, the male patients had a higher UA level (362.8 ± 96.0 vs. 304.8 ± 87.5, respectively; *P* < 0.001), RUI (3.2 ± 0.8 vs. 2.7 ± 0.8, respectively; *P* < 0.001), drinking, smoking, creatinine level, and eGFR and antiplatelet drug use rate. Female patients were older (70.3 ± 12.0 vs. 65.0 ± 12.4, respectively, *P* < 0.001) and have higher occurrences of AF (17.3 vs. 11.5%, respectively, *P* = 0.024) and anticoagulant use than those of men (Table 1).

**Table 1:** Baseline characteristics of participants.

**Variables**	**Male (*n* = 497)**	**Female (*n* = 272)**	***P* value**
**Demographic**			
Mean age, y (SD)	65.0 (12.4)	70.3 (12.0)	< 0.001
**Medical history**			
Alcohol consumption, *n* (%)	251 (50.5%)	9 (3.3%)	< 0.001
Smoking, *n* (%)	353 (71%)	13 (4.8%)	< 0.001
Hypertension, *n* (%)	361 (72.6%)	199 (73.2%)	0.875
Diabetes mellitus, *n* (%)	152 (30.6%)	83 (30.5%)	0.984
Dyslipidemia, *n* (%)	97 (19.5%)	46 (16.9%)	0.375
Atrial fibrillation, *n* (%)	57 (11.5%)	47 (17.3%)	0.024
**Clinical features**			
Time from onset to Admission, h (SD)	14.1 (8.5)	14.0 (8.5)	0.584
Systolic blood pressure, mmHg (SD)	151.8 (23.7)	154.0 (24.4)	0.233
Diastolic blood pressure, mmHg (SD)	88.3 (16.1)	86.4 (14.5)	0.103
Admission NIHSS score, mean (SD)	4.6 (5.0)	5.0 (4.9)	0.404
**TOAST classification, n (%)**			0.038
Large-artery atherosclerosis	234 (47.1%)	110 (40.4%)	0.077
Small-artery occlusion	191 (38.4%)	100 (36.8%)	0.649
Cardio-embolism	53 (10.7%)	46 (16.9%)	0.013
Undetermined etiology	15 (3.0%)	10 (3.7%)	0.623
Other etiology	4 (0.8%)	6 (2.2%)	0.179
**Laboratorial index**			
Platelet count, *10^9^/L (SD)	193.6 (75.8)	198.9 (65.7)	0.354
APTT, s (SD)	26.1 (4.9)	25.9 (7.9)	0.808
Serum UA, μmol/L (SD)	362.8 (96.0)	304.8 (87.5)	< 0.001
RUI, mean (SD)	3.2 (0.8)	2.7 (0.8)	< 0.001
Serum creatinine, μmol/L (SD)	82.6 (32.8)	65.2 (20.1)	< 0.001
eGFR, mL/min/1.73 m^2^, (SD)	86.1 (21.0)	82.2 (19.4)	0.012
HbA1c, %, (SD)	6.8 (1.6)	6.8 (1.9)	0.556
LDL-C, μmol/L (SD)	2.9 (1.1)	3.0 (1.8)	0.148
**Radiological characteristics**			
Spontaneous HT, *n* (%)	18 (3.6%)	12 (4.4%)	0.589
Large hemispheric infarction, *n* (%)	60 (12.1%)	25 (9.2%)	0.223
**Treatment**			
Antiplatelet, *n* (%)	486 (97.8%)	253 (93.0%)	0.001
Anticoagulant, *n* (%)	45 (9.1%)	49 (18.0%)	< 0.001
Antihypertensive, *n* (%)	272 (54.7%)	135 (49.6%)	0.176
Antidiabetic, *n* (%)	140 (28.2%)	72 (26.5%)	0.614

HT, hemorrhagic transformation; NIHSS, National Institutes of Health Stroke Scale; TOAST, Trial of ORG 10172 in Acute Stroke; APTT, activated partial thromboplastin time; UA, uric acid; IQR, inter-quartile range; RUI, the ratio of UA and its IQR (RUI=UA/IQR); eGFR, estimated glomerular filtration rate; HbA1c, hemoglobin A1c; LDL-C, low-density lipoprotein cholesterol; SD, standard deviation.

The male patients with spontaneous HT tended to have higher UA levels (428.3 ± 124.5 vs. 360.3 ± 94.0, respectively, P =0.003), RUI (3.7 ± 1.1 vs. 3.1± 0.8, respectively, *P* = 0.003), age, admission NIHSS score, higher occurrence of AF and LHI, and shorter time from the onset to admission compared to patients without. However, there was no significant association between UA/RUI and spontaneous HT in the female patients (*P* = 0.336) (Table 2).

**Table 2:** Univariate analysis to identify risk factors of spontaneous HT.

**Variables**	**Male (*****n*** = **497)**			**Female (*****n*** = **272)**		
	**With HT**	**Without HT**	***P*** **value**	**With HT**	**Without HT**	***P*** **value**
**Demographic**						
Mean age, y (SD)	71.3 (13.8)	64.8 (12.3)	0.028	73.5 (14.5)	70.2 (11.9)	0.451
**Medical history**						
Alcohol consumption, *n* (%)	10 (55.6%)	241 (50.3%)	0.662	0 (0.0%)	9 (3.5%)	1.000
Smoking, *n* (%)	13 (72.2%)	340 (71.0%)	0.909	0 (0.0%)	13 (5.0%)	1.000
Hypertension, *n* (%)	14 (77.8%)	347 (72.4%)	0.790	9 (75.0%)	190 (73.1%)	1.000
Diabetes mellitus, *n* (%)	9 (50.0%)	143 (29.9%)	0.690	4 (33.3%)	79 (30.4%)	0.760
Dyslipidemia, *n* (%)	6 (33.3%)	91 (19.0%)	0.136	2 (16.7%)	44 (16.9%)	1.000
Atrial fibrillation, *n* (%)	6 (33.3%)	51 (10.6%)	0.011	8 (66.7%)	39 (15.0%)	< 0.001
**Clinical features**						
Time from onset to admission, h (SD)	8.5 (8.0)	14.3 (8.5)	0.007	9.8 (9.2)	14.1 (8.4)	0.082
Systolic blood pressure, mmHg (SD)	148.8 (22.5)	151.9 (23.8)	0.586	161.7 (29.9)	153.6 (24.1)	0.265
Diastolic blood pressure, mmHg (SD)	83.9 (13.3)	88.5 (16.1)	0.236	85.9 (11.1)	86.4 (14.7)	0.901
Admission NIHSS score, mean (SD)	11.8 (8.1)	4.4 (4.6)	0.001	10.3 (6.8)	4.7 (4.7)	0.015
**TOAST classification**, ***n*** **(%)**			< 0.001			0.023
Large-artery atherosclerosis	9 (50.0%)	225 (47.0%)	0.801	8 (66.7%)	102 (39.2%)	0.073
Small-artery occlusion	0 (0.0%)	191 (39.9%)	0.001	0 (0.0%)	100 (38.5%)	0.005
Cardio-embolism	7 (38.9%)	46 (9.6%)	0.001	4 (33.3%)	42 (16.2%)	0.126
Undetermined etiology	2 (11.1%)	13 (2.7%)	0.098	0 (0.0%)	10 (3.8%)	1.000
Other etiology	0 (0.0%)	4 (0.8%)	1.000	0 (0.0%)	6 (2.3%)	1.000
**Laboratorial index**						
Platelet count, *10^9^/L (SD)	179.7 (70.6)	194.2 (76.0)	0.438	178.5 (72.4)	199.8 (65.4)	0.294
APTT, s (SD)	25.7 (3.5)	26.02 (4.9)	0.795	27.0 (5.2)	25.9 (8.0)	0.662
Serum UA, μmol/L (SD)	428.3 (124.5)	360.3 (94.0)	0.003	328.6 (57.5)	303.7 (88.6)	0.336
RUI, mean (SD)	3.7 (1.1)	3.1 (0.8)	0.003	2.9 (0.5)	2.7 (0.8)	0.336
Serum creatinine, μmol/L(SD)	88.9 (36.6)	82.3 (32.7)	0.401	68.6 (16.6)	65.0 (20.9)	0.550
eGFR, mL/min/1.73 m^2^, (SD)	77.7 (25.7)	86.4 (20.8)	0.086	76.7 (18.6)	82.4 (19.4)	0.320
HbA1c, %,(SD)	7.2 (2.3)	6.7 (1.6)	0.158	5.6 (2.1)	6,8 (1.9)	0.050
LDL-C, μmol/L (SD)	2.7 (0.8)	2.9 (1.1)	0.597	3.1 (1.1)	3.0 (1.8)	0.860
**Radiological characteristics**						
Large hemispheric infarction, *n* (%)	10 (55.6%)	50 (10.4%)	< 0.001	9 (75%)	16 (6.2%)	< 0.001
**Treatment**						
Antiplatelet, *n* (%)	18 (100%)	468 (97.7%)	1.000	10 (83.3%)	243 (93.5%)	0.201
Anticoagulant, *n* (%)	4 (22.2%)	41 (8.6%)	0.700	1 (8.3%)	48 (18.5%)	0.700
Antihypertensive, *n* (%)	8 (44.4%)	264 (55.1%)	0.372	4 (33.3%)	131 (50.4%)	0.248
Antidiabetic, *n* (%)	8 (44.4%)	132 (27.6%)	0.118	4 (33.3%)	68 (26.2%)	0.524

NIHSS, National Institutes of Health Stroke Scale; TOAST, Trial of ORG 10172 in Acute Stroke; APTT, activated partial thromboplastin time; UA, uric acid; IQR, inter-quartile range; RUI, the ratio of UA and its IQR (RUI=UA/IQR); eGFR, estimated glomerular filtration rate; HbA1c, hemoglobin A1c; LDL, low-density lipoprotein cholesterol; SD, standard deviation.

**Figure 2:**
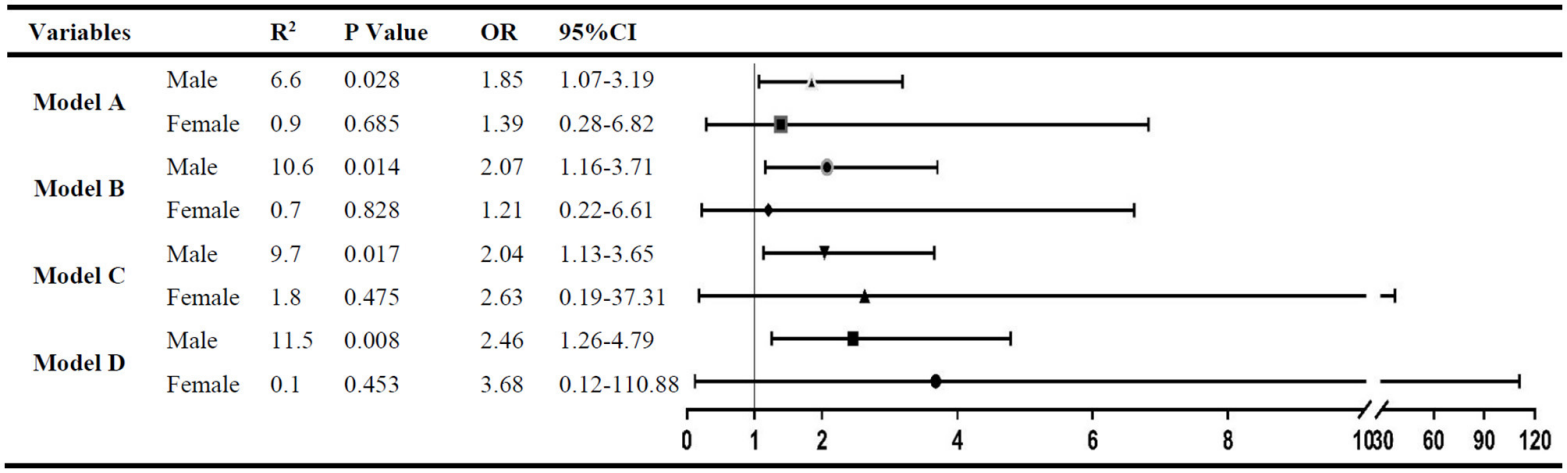
The multivariate analysis to identify the association between RUI and spontaneous HT. Variables adjusted in logistic regression models: Model A. Factors with a *P* < 0.05 in male (or female) subgroup univariate analysis were included; model B: factors with a *P* < 0.05 in both subgroup univariate analysis were included; model C: variables in model B plus antiplatelet treatment and anticoagulant treatment; and model D: variables in model C plus smoking, alcohol consumption, systolic blood pressure, and eGFR.

After adjustment for factors with a *P* < 0.05 (model A) in the univariate analysis of each group by logistic regression, the ratio of UA/IQR was found to be independently associated with spontaneous HT in male patients (OR: 1.85; 95% CI: 1.07–3.19; *P* = 0.028), but not in female patients (OR: 1.39; 95% CI: 0.28–6.82; *P* = 0.685). Furthermore, in the other three multivariate logistic regression models, the statistical results were consistent with model A after being adjusted for the factors with a *P* < 0.05 in the univariate analyses of both subgroups (model B), anticoagulant use and antiplatelet drug (model C), and smoking, alcohol consumption, SBP, and eGFR (model D) (Figure 2).

## Discussion

In this study, the RUI was independently associated with spontaneous HT in male patients admitted within 24 h after the onset, and the incidence of spontaneous HT increased by 85.0% for each IQR increase in the UA level. Interestingly, no similar association between the UA level and spontaneous HT was found in female patients.

Furthermore, we reported that UA levels were associated with spontaneous HT in male patients with acute ischemic stroke. However, this association was not found in female patients. UA levels are commonly available in medical settings, and the results of our study suggested that UA may be a potential target for interventions. Several previous studies have investigated the sex differences of UA in patients with cerebrovascular diseases (19, 20). Recently, a similar study reported that the incidence of spontaneous HT was higher in patients with low UA levels than in patients with high UA levels (13). They included 1,230 patients who received reperfusion therapy within 7 days from the onset of the symptoms. However, in the context of our study, this finding was not confirmed. The reason for these contradictory results may partly be due to inclusion criteria. Moreover, it has been proven that the UA level of patients with stroke decreased gradually within 7 days after the onset, but there was no significant difference between the UA level measured 24 h after admission (15). This also may reflex the dual effect of UA. In a cross-sectional study of 2,686 patients, Jeong et al. reported that a high UA level was a risk factor for cerebral microbleeds only in male patients (19), and we confirmed and extended this finding in our study. Further studies are needed to explore whether these patients are potential candidates for interventions.

In previous studies, reperfusion treatment is one of the mechanisms of spontaneous HT, subsequently affecting the outcome. Previous studies regarding the relationship between UA and spontaneous HT have shown conflicting results in the thrombolysis population and non-thrombolysis group. Thrombectomy, the restoration of blood flow to the salvageable ischemic brain tissue, is consistent with the aforementioned mechanism of spontaneous HT, and a higher incidence of spontaneous HT was reported in previous studies. The reason for these contradictory results may partly be due to the modifying effect of reperfusion strategies on spontaneous HT in these studies. Thus, we excluded those patients from this study.

The exact underlying mechanism of UA levels on spontaneous HT remains unknown. Generally, UA is an abundant antioxidant in humans and is supposed to play a protective role in cardio-cerebral vascular diseases. The possible explanation of sex-dependent differences in UA levels on spontaneous HT was the uricosuric effect of estrogen (21), the inhibition of oxidative stress of blood vessels by estrogen (22), and the redox shuttle mechanism of UA (23). These three factors result in higher UA and lower antioxidant capacity in male patients than in female patients. In addition, UA is more effective in promoting oxidation in an environment with relatively lower-antioxidative substances. Therefore, the stronger oxidation-promoting property of UA in male patients may be responsible for the sex difference in the occurrence of spontaneous HT. However, the opposite result has been found in many large-scale clinical studies (24–26). A literature review revealed that UA, which carries over half of the antioxidant capacity in plasma, may be involved in spontaneous HT through oxidative stress (4). This involvement can be partly explained by the following reasons: first, the production of UA by xanthine oxidase itself produces oxygen free radicals (27) and second, more oxygen free radicals will be produced after ischemia–reperfusion (5). UA has a redox shuttle effect in which the presentation of the antioxidant or pro-oxidant properties of UA depends on the surrounding environment. Specifically, antioxidant activity occurs when antioxidant substances are abundant, and pro-oxidant activity occurs if there are more pro-oxidants (23). In the environment of more oxygen free radicals in the ischemia–reperfusion tissue, UA tends to be pro-oxidative. Therefore, UA may further aggravate oxidative stress and increase blood–brain barrier damage through the aforementioned mechanisms, which leads to spontaneous HT.

It should be noted that our study had some limitations. First, it was a single-center retrospective study with a relatively small sample size. The impact of UA on spontaneous HT seems to be limited in the sex-specific subgroups, and this clinical relevance may not be generalizable to patients with reperfusion treatment. In addition, a multicenter prospective study with a large sample size is required to further confirm and explore the association between UA and the subtypes of HT. Second, UA levels have been found to change over time in patients with stroke (15), whereas our study enrolled patients admitted to the hospital within 24 h after the stroke onset only. Hence, there will be a limited scope of application in terms of the findings in our study. In addition, our study retrospectively explained the association between the single UA level and spontaneous HT at admission, so it is still necessary to further clarify such a relationship by dynamical examination of the UA level in a prospective study.

## Conclusion

In conclusion, among the non-reperfusion patients with acute ischemic stroke within 24 h after admission, the level of UA was independently and positively associated with the occurrence of spontaneous HT in male patients. More prospective research is needed to confirm these results.

## Data availability statement

The raw data supporting the conclusions of this article will be made available by the authors, without undue reservation.

## Ethics statement

The studies involving human participants were reviewed and approved by the Ethics Board of the First Affiliated Hospital of Chongqing Medical University. The patients/participants provided their written informed consent to participate in this study.

## Author contributions

YT and M-SL: study concept and design. YT, M-SL, and CF: acquisition of data. YT: statistical analysis and drafting of the manuscript. G-QL: critical revision of the manuscript for important intellectual content and study supervision. Analysis and interpretation of data were done by all authors. All authors contributed to the article and approved the submitted version.
